# Do patient-reported outcome measures cover personal factors important to people with rheumatoid arthritis? A mixed methods design using the International Classification of Functioning, Disability and Health as frame of reference

**DOI:** 10.1186/s12955-015-0214-8

**Published:** 2015-02-25

**Authors:** Mona Dür, Michaela Coenen, Michaela Alexandra Stoffer, Veronika Fialka-Moser, Alexandra Kautzky-Willer, Ingvild Kjeken, Răzvan Gabriel Drăgoi, Malin Mattsson, Carina Boström, Josef Smolen, Tanja Alexandra Stamm

**Affiliations:** Medical University of Vienna, Department of Internal Medicine III, Division of Rheumatology, Währinger Gürtel 18-20, 1090 Vienna, Austria; IMC University of Applied Sciences Krems, Department of Health Sciences, Occupational Therapy, Piaristengasse 1, 3500 Krems, Austria; Ludwig-Maximilians-University, Department of Medical Informatics, Biometry and Epidemiology, Research Unit for Bio Psychosocial Health, Marchioninistraße 17, 81377 Munich, Germany; Medical University of Vienna, Department of Physical Medicine and Rehabilitation, Währinger Gürtel 18-20, 1090 Vienna, Austria; Medical University of Vienna, Department of Internal Medicine III, Division of Diabetology, Währinger Gürtel 18-20, 1090 Vienna, Austria; Diakonhjemmet Hospital, National Advisory Unit on Rehabilitation in Rheumatology, Department of Rheumatology, Postbox 23 Vinderen, 0319 Oslo, Norway; “Victor Babeş” University of Medicine and Pharmacy, Department of Rehabilitation, Physical Medicine and Rheumatology, Piata Eftimie Murgu 2, Timişoara, 300041 Timis Romania; Luleå University of Technology, Department of Health Sciences, SE-971 87, Luleå, Sweden; Sunderby Hospital, Department of Physiotherapy, SE-971 80, Luleå, Sweden; Karolinska Institutet, Department of Neurobiology, Care sciences and Society, Division of Physiotherapy, Alfred Nobels Allé 23, 141 83 Huddinge, Stockholm, Sweden; Fachhochschule Campus Wien, University of Applied Sciences, Department of Health, Favoritenstraße 226, 1100 Vienna, Austria

**Keywords:** Qualitative research methods, Health promotion, Outcome research, Patient perspective, Rehabilitation

## Abstract

**Background:**

Personal factors (*PFs*) are internal factors that determine functioning and the individuals’ experience of disability. Their coverage by patient-reported outcome measures (PROMs) has not been examined in rheumatoid arthritis (RA) so far. The aims of this study were to identify *PFs* important in the life stories of people with RA and to determine their coverage by PROMs used in RA.

**Methods:**

The qualitative data of people with RA was explored to identify *PFs*. Additionally a systematic literature search was conducted to find PROMs used in RA. PROMs items were linked to the components, domains and categories of the International Classification of Functioning, Disability and Health (ICF) to determine the coverage of important *PFs* by PROMs.

**Results:**

Twelve *PFs* were found to be important in the life stories of people with RA. The *PFs coping* and *reflecting about one’s life in an optimistic way* were covered most frequently, each by 14 of the 42 explored PROMs, while *job satisfaction* was not covered at all. The *London Coping with Rheumatoid Arthritis Questionnaire, General Self-Efficacy Scale, Arthritis Self-Efficacy Scale*, *Rheumatoid Arthritis Self-Efficacy Questionnaire* and *Revised Ways of Coping Inventory* covered most *PFs*. Nineteen PROMs did not cover any of the *PFs*.

**Conclusion:**

Several *PFs* were identified as important in the life stories of people with RA, but only 55% of the PROMS covered some of these *PFs*. When evaluating *PFs* important to people with RA, health professionals should be alert on which PROMs can be used to assess which *PFs*.

## Background

Rheumatoid arthritis (RA) is a chronic autoimmune disease, characterized by joint inflammation, pain, joint swelling, morning stiffness, and fatigue which may lead to loss of functioning in daily life [1]. The prevalence ranges from 0,5-2% and is 3 - 4 times higher in women than in men [2]. However, the current understanding of the burden of the disease comprises not only clinical symptoms, but also other aspects that have an impact on living with RA, including environmental factors and personal factors, such as people’s social support or self-efficacy [3-5].

The International Classification of Functioning, Disability and Health (ICF) [6] is a common conceptual framework used to understand, describe and measure the dimensions of human functioning, disability and health [7]. Within the ICF, an individual’s functioning is conceptualized as result of the interplay between body functions and structures, activities and participation and contextual factors that include environmental and personal factors (*PFs*) [6].

*PFs* have played a tangential part in relation to ICF-based health outcome research. In the ICF, *PFs* are defined as internal factors that determine functioning and the individuals’ experience of disability. *PFs* comprise “features of the individual” such as coping, social background and psychological factors impacting health outcomes [6]. However, even if a few researchers have explored *PFs* through consensus processes [8], systematic reviews [9] or qualitative interviews with patients [10], they are not yet classified according to the ICF “taxonomy” [6,7].

For example, the RA ICF core set was developed to provide a set of categories that best describes the problems of functioning of people with RA [11]. Within three validation studies based on qualitative data several *PFs* were identified as meaningful which have not been covered by the ICF core sets [12-15]. Further, even though single *PFs* have been explored in people with RA [16,17], they have been left out in the examination of the coverage of the perspectives of patients with RA by patient-reported outcome measures (PROMs), as it has been done in other chronic diseases [18-20]. Thus, it is unclear how PROMs cover *PFs* important to people with RA. Additionally, *PFs* and their meaning to people with RA may change over time and the course of disease [21,22]. Hence, they need to be explored within a long-term perspective over the life course.

Furthermore, the new and effective biologic therapies facilitate the inclusion of other important aspects such as *PFs* as targets of non-pharmacological treatment of people with RA [6,23,24]. For example, interventions targeting *PFs,* such as coping strategies or medication beliefs, could support individuals to achieve their fullest potential on functioning, to reduce the impact of RA [24,25], and to increase medication adherence [26]. To assess the need for or to evaluate non-pharmacological treatment in clinical practice or rehabilitation targeting *PFs*, health professionals and researchers should be alert on which PROMs cover which *PFs*.

The aims of this study were to identify *PFs* important in the life stories of people with RA and to determine their coverage by PROMs used in RA.

## Methods

We used a mixed methods design consisting of a qualitative analysis, a systematic literature search and a linking process. The current project was part of a larger study [27,28].

### Exploration of qualitative data to identify personal factors important in the life stories of people with RA

**Figure 1:**
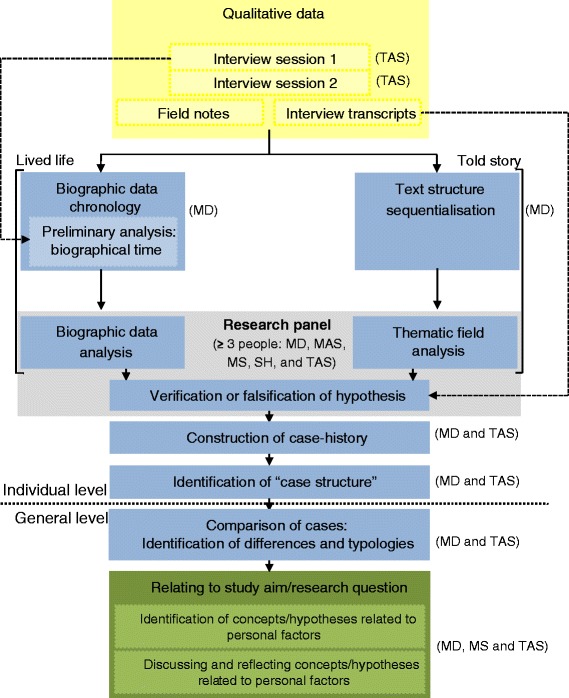
**Flow diagram of the data collection and analysis following the BNIM.** (Initials) = researcher(s) who conducted the respective step of the BNIM.

Firstly, a secondary analysis of qualitative data of a previous study [29,30] was conducted. In the respective study, patients from the rheumatologic outpatient clinic of the Medical University of Vienna, Austria, diagnosed with RA [31] were asked for participation. A small sample size of 15 participants with a diverse range was aimed to gather rich and meaningful data [32]. Recruitment used a maximum variation sampling strategy [33] in terms of sex, age, former professional status and disease duration. Inclusion criteria were “being early retired” at the time of the interviews, having past employment experience (≥20 hours per week), no history of psychiatric and/or other neuro-motor disease and German as first language. Since we were interested in the identification of *PFs* which could be complex, such as coping or resilience, we decided to use people’s life stories and to follow the biographical narrative interpretative method (BNIM) [33,34]. In accordance to the study aim the interviews’ verbatim transcripts of the life stories were used to determine *PFs* which were important over the life and disease course of people with RA. Therefore, each transcript was analyzed by two researchers independently (MD, MS, and TAS). In case of disagreement, each case was discussed in a research panel of three people who together made a final informed decision, whether or not a certain *PF* was encompassed in the respective life story. *PFs* which were found among different life stories were identified based on the exploration of people’s interpretation of their life’s experience and their biography [34] and used for the exploration of their coverage by PROMs. A flow chart of the different steps of the BNIM is depicted in Figure 1. For detailed information we refer to further literature [33,34].

### Systematic literature search to find PROMs used in RA

Secondly, we conducted a systematic literature search in 2013 using PubMed, CINAHL and PsycInfo to find PROMs used in RA and to extract their items. The following combination of keywords was used to search the articles: [(rheumatoid arthritis)] AND [(outcome) OR (assessment) OR (instrument) OR (measure) OR (questionnaire)] AND [(self-reported) OR (patient-reported) OR (patient perspective)]. For inclusion, articles had to be written in English and published in a peer-reviewed journal and the description of the use or development of at least one PROM had to be contained in the title or the abstract. Candidate articles were independently reviewed by two researchers (MD and AB) using a data extraction form, to identify the descriptions of PROMs. A PROM was included when the following criteria were applied: assessing functioning and/or functional health and/or those *PFs* which were identified in the qualitative analyses. PROMs items which were not provided within these articles were obtained from reference checking or on request from their authors. PROMs specifically designed for children or adolescents and single-VAS-assessments for disease activity of RA were excluded.

### Linking process to determine the coverage of important personal factors by PROMs

**Figure 2:**
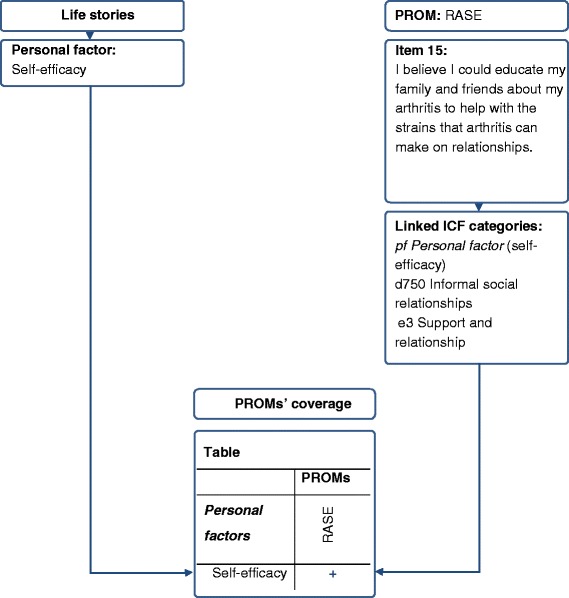
**Example: Linking process to determine the coverage of important personal factors by patient-reported outcome measures.** Comparison of one personal factor to one linked item of the Rheumatoid Self-efficacy Questionnaire; Abbreviations: ICF = International Classification of Functioning, Disability and Health, PROMs = Patient-reported outcome measures, RASE = Rheumatoid Self-efficacy Questionnaire.

Finally, we determined which *PFs* were covered by which PROMs using the ICF [6] as reference. Items of the PROMs were linked to ICF categories by two researchers (MD and MC). In case of disagreement an informed decision was made by one further researcher skilled and experienced in the ICF linking process (TAS). The linking process followed a standard procedure by the use of the current ICF-linking rules [35]. Concerning the complexity of certain items, we applied the ICF-linking rule referring to items encompassing different constructs, an example is shown in Figure 2. Articles providing ICF categories linked to the selected PROMs were used.

The linked ICF categories of each PROM were compared to the *PFs* from the qualitative data by mapping them to each other. Finally, PROMs were explored in order to report how many PROMs were available to assess each *PF* and those *PFs* for which no PROM existed. An example is given in Figure 2.

### Ethical considerations

Participants received information about study procedures and ethical considerations and gave written and oral informed consents. Confidentiality was guaranteed and names were changed in the given examples. The study was approved by the ethics committee of the Medical University of Vienna, Austria.

## Results

### Personal factors important in the life stories of people with RA

For the current study, we used the data of 15 people with RA, 11 women and 4 men with a median age of 54 years and disease duration of 11 years [29,30]. Demographic data is depicted in Table 1.

**Table 1:** **Demographic data of the participants**

	**Women**	**Men**
*n* (%) Total	11 (73)	4 (27)
*n* (%) International Standard Classification of Education (ISCED) level 3: completed vocational education or secondary education premising the access to higher education	6 (55)	3 (75)
Median Age (interquartile range)	52 (43–61)	56 (42–58)
Median Disease duration (interquartile range)	10 (8–20)	14 (10–26)

In the secondary analysis of the life stories of people with RA the following 12 *PFs* were identified as being important: *Adaptation to changed living conditions*; *coping*; *eating habits and weight concerns*; *involvement into disease management*; *job satisfaction*; *meaningful activities for the individual and/or the societal context*; *own attitudes*; *reflecting about one’s life in an optimistic way*; *resilience*; *self-efficacy*; *sense of coherence* and *social appreciation*.

In the following section, we give two examples of important *PFs*: In the life story of Hans, a 58 years old varnisher, we identified *adaptation to changed living conditions.* Hans did not get a job after he had left the previous one. Thus, his life story contained several descriptions on changes which were adaptations to living with RA:

*“Depressing when you suddenly become useless at the age of 40, not knowing how life will go on and how to get oneself and one’s family. So, my wife worked half time and I took care of the children, as far as that was possible”.* (First interview, lines 51–53)

Another example is Maria, a 42 years old woman. She did not let the disease “rule” her life and supported others to care about their health and wellbeing. Finally, Maria became an “advocate” for people with RA. We identified *self-efficacy* and *involvement into disease management* when she told about her engagement in acquiring knowledge and skills.

*“What I wanted to know was how to handle it* [the disease]? *So, I asked my physician* [rheumatologist] *to prepare me for the case that the worst happens and we talked it through. I have written down everything. In the case I found myself in troubles, I looked through my notes and could help myself”.* (First interview, lines 537–546)

*“My ambition spurred me on, not to accept everything related to the disease and to let it rule my life. I have bought medical books, attended specialist conferences* [on rheumatic diseases], *I went to libraries and studied* [RA specific] *drugs and their side effects. I started to understand the physician a little when he talked about the medication. I felt that I could have a determining influence on the decision which drug should be tried next”.* (First interview, lines 687–701) *“I realized that meanwhile I was engaged in the management of my disease to the same extent as I was engaged in my job formerly”.* (First interview, lines 719–721)

Three *PFs* were found to be important in the life stories of women only. These were *reflecting about one’s life in an optimistic way*, *involvement into disease management* and *job satisfaction.* While *coping* and *meaningful activities for the individual and/or the social context* was important in the life story of all men, the same was true for *own attitudes* in women. The frequency and percentage of identified *PFs* per sex are depicted in Table 2.

**Table 2:** **Frequency of personal factors per sex**

**R**	**Personal factors**	***n*** **(%)**	**f (%)**	**m (%)**
**1**	Own attitudes	13 (87)	11 (100)	2 (50)
**2**	Adaptation to changed living conditions	11 (73)	9 (82)	2 (50)
**3**	Meaningful activities for the individual and/or the social context	12 (80)	8 (73)	4 (100)
**4**	Eating habits and weight concerns	10 (67)	8 (73)	2 (50)
**5**	Coping	9 (60)	5 (45)	4 (100)
**6**	Reflecting about one’s life in an optimistic way	7 (47)	7 (64)	0
**6**	Involvement into disease management	7 (47)	7 (64)	0
**6**	Self-efficacy	7 (47)	6 (55)	1 (25)
**7**	Sense of Coherence	6 (40)	4 (36)	2 (50)
**8**	Job satisfaction	5 (33)	5 (45)	0
**9**	Social appreciation	4 (27)	3 (27)	1 (25)
**9**	Resilience	4 (27)	2 (18)	2 (50)

Abbr.: R = Rank, n = number, f = female, m = male.

### PROMs used in RA

**Figure 3:**
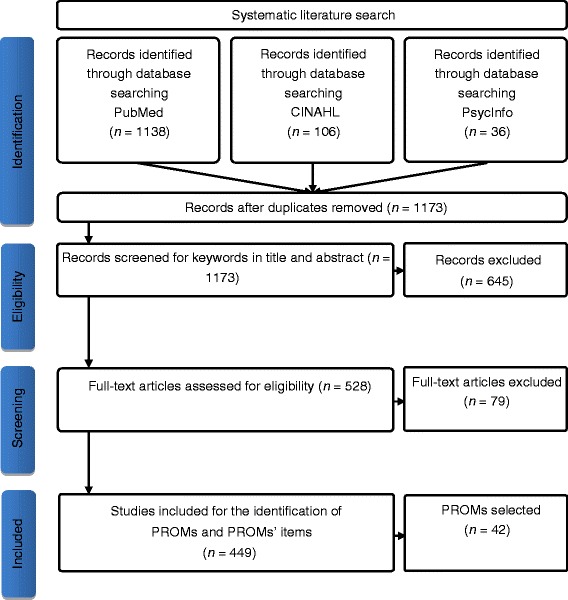
**Flow diagram.**

The systematic literature search resulted in 1280 hits, of which 831 were excluded due to 107 duplicates and 724 irrelevant articles. Finally, 449 articles were used to identify the PROMs used in RA, as described in Figure 3.

In total forty-two PROMs met our inclusion criteria. They are listed and described in Table 3.

**Table 3:** Characteristics of the identified patient-reported outcome measures

**Abbreviations**	**Names of patient-reported outcome measures**	**Items**
AIMS2-SF	Arthritis Impact Measurement Scales Short Form [36]	26
APaQ	Activity Participation Questionnaire [37]	2
ASES	Arthritis Self-Efficacy Scale [38]	20
B-WOC-R	Brief Revised Ways of coping inventory [39]	18
BRAF MDQ	Bristol Arthritis Fatigue Multi-Dimensional Questionnaire [40]	20
BRAF NRS	Bristol Arthritis Fatigue Numerical Rating Scale [40]	3
CFS	Chalder Fatigue Scale [41]	11
CIS 20R	Checklist Individual Strength [42]	20
CIS 8R	Checklist Individual Strength [42]	8
C-RAQ	Coping with Rheumatoid Arthritis Questionnaire [25]	20
DRP	Disease Repercussion Profile [43]	6
EC-17	Effective Musculoskeletal Consumer Scale (Short Form) [44]	17
EQ-5D	EuroQuoL Health questionnaire [45]	5
FACIT-F	Functional Assessment Chronic Illness Therapy (Fatigue) [46]	13
FSS	Fatigue Severity Scale [47]	9
GSES	General Self-Efficacy Scale [48,49]	10
HADS	Hospital Anxiety and Depression Scale [50]	14
HAQ	Health Assessment Questionnaire [51]	20
HAQ-II	Health assessment questionnaire ii [52]	10
HAQ-DI	Health Assessment Questionnaire Disability Index [53]	20
JP SES	Joint Protection Self-efficacy Scale [54]	10
LCRAQ	London Coping with Rheumatoid Arthritis Questionnaire [55]	36
LOT-R	Life Orientation Test-Revised [56]	8
MAF	Multi-dimensional Assessment of Fatigue [57]	15
MFI	Multi-dimensional Fatigue Inventory [58]	20
MHAQ	Modified Health Assessment Questionnaire [59]	8
MHLC - C	Multidimensional Health Locus of Control C-Form [60]	18
PI-HAQ	Personal Impact Health Assessment Questionnaire [61]	20
PRO-CLARA	Patient Reported Outcome - Clinical Arthritis Activity [62]	21
RAID	Rheumatoid Arthritis Impact of Disease score [63]	12
RAPID 3	Routine Assessment of Patient Index Data [64]	24
RAQoL	Rheumatoid Arthritis Quality of life [65]	30
ROAD	Recent-Onset Arthritis Disability Index [66]	12
RASE	Rheumatoid Arthritis Self-Efficacy Questionnaire [67]	28
SACRAH	Score for Assessment & Quantification of Chronic Rheumatoid Affections of the Hands [68]	23
SF-36	Short-Form Health Survey 36-item [69,70]	36
SOC-13	Sense of Coherence scale-13 [71]	13
SSQS & SQT	Social Support Questionnaire Transactions & Satisfaction with supportive transactions [72]	46
SSS	MOS Social Support Survey [73]	20
WOC-R	Revised Ways of Coping Inventory [74]	50
10 ADLMDHAQ	10 Activities of Daily Living Multidimensional Health Assessment Questionnaire [75]	10
14 ADLMDHAQ	14 Activities of Daily Living Multidimensional Health Assessment Questionnaire [75]	14

### PROMs coverage of important personal factors

The ICF categories linked to the items of eight PROMs [36,45,51,59,64,68,70,76] were used from existing literature [28,77,78]. The mapping of *PFs* to the PROMs is depicted in Table 4.

**Table 4:** **Coverage of personal factors by patient-reported outcome measures**

	**Important personal factors**			
**PROMs**	***Adaptation to changed living conditions***	***Coping***	***Eating habits & weight concerns***	***Involvement into disease management***
**AIMS2-SF**				
**APaQ**				
**ASES**		+		+
**B-WOC-R**		+		
**BRAF MDQ**				
**BRAF NRS**		+		
**CFQ**				
**CIS 20R**				
**CIS 8R**				
**C-RAQ**		+		
**DRP**				
**EC-17**		+		+
**EQ-5D**				
**FACIT-F**		+		
**FSS**				
**GSES**		+		
**HADS**				
**HAQ**				
**HAQ-II**				
**HAQ-DI**				
**JP SES**				
**LCRAQ**	+	+	+	+
**LOT-R**				
**MAF**				
**MFI**				
**MHAQ**				
**MHLC-C**		+		
**PI-HAQ**				
**PRO-CLARA**				
**RAID**		+		+
**RAPID 3**		+		
**RASE**		+		
**RAQoL**				
**ROAD**				
**SACRAH**				
**SF-36**				
**SOC-13**				
**SSQT & -S**				
**SSS**				
**WOC-R**		+		+
**10 ADLMDHAQ**				
**14 ADLMDHAQ**		+		
	**Important personal factors**			
**PROMs**	***Job satisfaction***	***Meaningful activities for the individual/the social context***	***Own attitudes***	***Reflecting about one’s life in an optimistic way***
**AIMS2-SF**				
**APaQ**				
**ASES**			+	
**B-WOC-R**				+
**BRAF MDQ**				
**BRAF NRS**				
**CFQ**				
**CIS 20R**				
**CIS 8R**				
**C-RAQ**				+
**DRP**				+
**EC-17**				+
**EQ-5D**				
**FACIT-F**		+		+
**FSS**				
**GSES**			+	+
**HADS**				+
**HAQ**				
**HAQ-II**				
**HAQ-DI**				
**JP SES**				
**LCRAQ**			+	+
**LOT-R**				+
**MAF**				
**MFI**				
**MHAQ**				
**MHLC-C**			+	+
**PI-HAQ**				
**PRO-CLARA**				
**RAID**				
**RAPID 3**				
**RASE**			+	+
**RAQoL**				+
**ROAD**				
**SACRAH**				
**SF-36**			+	
**SOC-13**		+		+
**SSQT & -S**				
**SSS**				
**WOC-R**				+
**10 ADLMDHAQ**				
**14 ADLMDHAQ**				
	**Important personal factors**			
**PROMs**	***Resilience***	***Self-efficacy***	***Sense of coherence***	***Social appreciation***
**AIMS2-SF**				
**APaQ**				
**ASES**		+	+	
**B-WOC-R**	+	+		
**BRAF MDQ**				
**BRAF NRS**				
**CFQ**				
**CIS 20R**				
**CIS 8R**				
**C-RAQ**	+	+		
**DRP**				
**EC-17**		+		
**EQ-5D**				
**FACIT-F**	+			
**FSS**				
**GSES**	+	+	+	
**HADS**	+			
**HAQ**				
**HAQ-II**				
**HAQ-DI**				
**JP SES**		+		
**LCRAQ**	+	+		+
**LOT-R**	+		+	
**MAF**	+			
**MFI**		+		
**MHAQ**				
**MHLC-C**		+		
**PI-HAQ**				
**PRO-CLARA**				
**RAID**				
**RAPID 3**	+			
**RASE**	+	+		
**RAQoL**		+		
**ROAD**				
**SACRAH**				
**SF-36**				
**SOC-13**			+	
**SSQT & -S**				
**SSS**				
**WOC-R**	+	+		
**10 ADLMDHAQ**				
**14 ADLMDHAQ**				

PROMs = abbreviated names of patient-reported outcome measures; + = personal factor is covered by the specific patient-reported outcome measures.

The *PFs coping* and *reflecting about life in an optimistic way* were covered most frequently (each by 14 PROMs), followed by *resilience* and *self-efficacy* (each by 12 PROMs). Compared to that, *job satisfaction* was not covered by any of the PROMs. The *PF own attitudes* was covered by six, *involvement into disease management* by five, *sense of coherence* by four and *meaningful activities* by two PROMs. *Adaptation to changed living conditions*, *social appreciation* and *eating habits and weight concerns* were covered once, each by the London Coping with Rheumatoid Arthritis Questionnaire (LCRAQ) [79].

The LCRAQ covered most (nine) *PFs, followed by the General Self-Efficacy Scale* (GSES) [48,49] which covered six *PFs*. The *Arthritis Self-Efficacy Scale* (ASES) [38], the *Rheumatoid Arthritis Self-Efficacy Questionnaire* (RASE) [67] and the *Revised Ways of Coping Inventory* (WOC-R) [74] captured five *PFs* each. Nineteen of the 42 explored PROMs covered no *PF*, including the different versions of the *Health Assessment Questionnaire* (HAQ) except the 14 Activities of Daily Living Multidimensional HAQ (14 ADLMDHAQ) [51-53,61,75], as shown in Table 4.

## Discussion

In the current study we identified 12 *PFs* being important in the life stories of people with RA and explored their coverage by 42 PROMs used in RA. The results of this study can support health professionals and researchers in their selection of which PROMs to use, when assessing the need for or evaluating the effect of non-pharmacological treatment in clinical practice or rehabilitation [80] targeting the identified *PFs*.

*PFs* which were found to be important to people with RA could get more emphasis in ICF-based health outcome research. For example, self-efficacy was found to facilitate the maintenance of physical activity [4] and to decrease fatigue [81], pain [82] and the development of cardiovascular risk [83,84]. Since cardiovascular diseases account for approximately 50% of mortality [85], the prevention of cardiovascular risk is an important target in the disease management of RA [86] including pharmacological and non-pharmacological methods [87]. Thus, health outcome research focusing on *PFs* with strong evidence for their health determining effect could be of great value to support individuals achieving their fullest functioning and health.

Due to unequal proportion of female and male participants, the identified gender differences of *PFs* need to be treated with caution. The findings could indicate a difference in the meaning of these *PFs* in the life stories between women and men with RA. Therefore, the selection of the outcomes and related PROMs should take into account potential gender differences to consider the preferences and values regarding *PFs* of women and men in the evaluation of health care interventions.

Even though, most of the explored PROMs were not designed to assess a range of *PFs*, one PROM was outstanding in its coverage of *PFs*: the LCRAQ which could be used to address most of the identified *PFs*. The GSES could be employed to measure six *PFs*. Additionally, the ASES, the RASE and the WOC-R were found to assess five different *PFs*, respectively. While the *PFs coping*, *reflecting about one’s life in an optimistic way* and *sense of coherence* could be assessed by all of these PROMs, *adaptation to changed living conditions*, *eating habits and weight concerns*, and *social appreciation* was found to be covered by the LCRAQ only. In addition, the PROMs which covered most of the *PFs*, could be used to measure the *PFs own attitudes* (except the WOC-R) and *resilience* (except the ASES). Consequently, one of these PROMs could be selected for the evaluation of health care interventions having regard to the different *PFs* important in the life stories of people with RA. However, for the use in clinical practice and research, other psychometric properties and applicability in different cultural contexts need to be considered.

Commonly used PROMs in clinically routine, e.g. the HAQ could be combined with others. Only the 14 ADLMDHAQ [75] covers *PFs* (*n* = 2)*,* while the other versions of the HAQ do not cover any of the *PFs*. In addition, it would be interesting to develop RA specific PROMs that address *adaptation to changed living conditions*, *social appreciation*, *eating habits and weight concerns*, *job satisfaction* and *meaningful activities*, since these *PFs* have rarely been addressed in the PROMs. In the clinical routine and rehabilitation, health professionals such as nurses, occupational therapists or physiotherapists, may use other assessments and thus, could address *PFs* to complement and/or to conduce to success of pharmacological treatment for people with RA [26].

This research had some limitations. An inclusion of PROMs published in various languages could give valuable information about their potential utilization to assess *PFs*. Another study could include *PFs* important to patients of younger age to determine their coverage of PROMs, based on previous research [88]. In addition, further studies could focus on the perspectives of patients of different cultural backgrounds or specific person groups such as parents or patients with recent onset. The so called member checking method could have contributed to the credibility of the findings. In the current project we explored the content validity of PROMs, referring to their coverage of concepts which are relevant to the target population [89]. Despite determining PROMs’ content validity, a critical appraisal of other measurement properties could have provided additional important information. However, we did not explore other psychometric properties of the selected PROMs since this was not the focus of our study. Additional studies are warranted to generalize the findings of the current project to other people with RA.

## Conclusion

Taken together, the identified *PFs* are important in the life stories of people with RA and could be addressed in clinical practice and rehabilitation by different health professionals in order to support their patients’ functioning and health. The LCRAQ, the GSES, the ASES, the RASE and the WOC-R could be used when assessing the need for or evaluating health care interventions targeting the identified *PFs* and thus contribute to an increasing benefit for people with RA. Furthermore the findings can be used for further development of existing PROMs and to guide their use in clinical practice, rehabilitation and research.

## Abbreviations

ASES: Arthritis self-efficacy scale
GSES: General self-efficacy scale
HAQ: Health assessment questionnaire
ICF: International classification of functioning, disability and health
LCRAQ: London coping with rheumatoid arthritis questionnaire
MDHAQ: Multidimensional health assessment questionnaire
PF (s): Personal factor (s)
PROM (s): Patient-reported outcome measure (s)
RA: Rheumatoid arthritis
RASE: The rheumatoid arthritis self-efficacy questionnaire
WOC-R: Revised ways of coping inventory
